# A risky business: Teaching clinical risk assessment in the midst of a global pandemic

**DOI:** 10.1192/j.eurpsy.2021.1590

**Published:** 2021-08-13

**Authors:** A. Oates, C. Wilson-Jones, S. Butler

**Affiliations:** Gkt School Of Medicine, Undergraduate Psychiatry, London, United Kingdom

**Keywords:** formulation, MedEd, psychiatry, risk

## Abstract

**Introduction:**

Assessing risk is an important core skill yet there is not a consensus as to how to teach it. Clinically, there has been a move away from using risk prediction tools in favour of clinical judgement.We describe an iterative process to develop high quality, online teaching around risk assessment for medical undergraduates.

**Objectives:**

To teach the clinical skill of risk assessment to enable medical students to evaluate and manage risk when encountering patients with mental health issues.

**Methods:**

A half day tutorial was designed and refined in an iterative process using feedback from participants on this session and other concurrent teaching occurring in the department. Sessions were also reviewed by external medical educators to ensure quality and learning objectives were met.

**Results:**

The average rating from 62 students was 4.4/5. Students commented that the session was well organised and delivered. Following feedback, the use of actors was prioritised to simulate evolving clinical situations. Students placed a high value on this: “simulated patients were amazing! They were really interesting and I was able to practice the skills I learnt over placement”. Logistical changes e.g. more breaks, followed appreciation of the exhausting nature of the session and maintained student engagement. There was increased emphasis on promoting group interaction through functions like a ‘break-out room’.

**Conclusions:**

This session may give educators confidence that they can take risks when teaching the skill of risk assessment. Students were receptive and meaningfully engaged with concepts such as clinical judgement and bio-psycho-social formulations as opposed to ‘tick box’ assessments.

